# Distúrbios do Sistema de Condução Atrioventricular e Potenciais Riscos de Eventos Arrítmicos em Atletas de Alta Resistência

**DOI:** 10.36660/abc.20200668

**Published:** 2020-07-28

**Authors:** Carlos Alberto Cordeiro Hossri

**Affiliations:** 1 Instituto Dante Pazzanese de Cardiologia São PauloSP Brasil Instituto Dante Pazzanese de Cardiologia,São Paulo, SP - Brasil; 2 Hospital do Coração São PauloSP Brasil Hospital do Coração, São Paulo, SP – Brasil; 3 HCor Associação Beneficiente Síria São PauloSP Brasil HCor - Associação Beneficiente Síria, São Paulo, SP - Brasil

**Keywords:** Atletas, Treinamento de Resistência, Aptidão Física, Remodelação Ventricular, Sedentarismo, Arritmias Cardíacas, Eletrocardiografia/métodos, Função Ventricular

*Minieditorial referente ao artigo: Avaliação do Tempo de Condução Atrioventricular Dinâmica para Acoplamento ao Intervalo RR em Atletas e Indivíduos Sedentários*

Algumas alterações eletrocardiográficas são comumente observadas em atletas de alta resistência ou alto rendimento e, por vezes, apresentam características semelhantes às observadas em indivíduos idosos ou com doença cardiovascular.^1 - 4^

O treinamento de elevada intensidade em atletas de alto rendimento pode induzir adaptações fisiológicas intrínsecas ao sistema de condução do estímulo cardíaco e, consequentemente, maior prevalência de anormalidades na condução atrioventricular (AV).^3 , 5^

Os mecanismos fisiológicos ou mesmo fisiopatológicos pelos quais o treinamento atlético induz tais alterações intrínsecas no sistema de condução cardíaca ainda apresentam entendimento limitado, e provavelmente devem ser multifatoriais. No entanto, as alterações anatômicas observadas, como dilatação atrial e ventricular, demonstraram a criação de um remodelamento mecânico-elétrico necessário para causar adaptações eletrofisiológicas AV intrínsecas.^4 - 6^

Dentre as expressões eletrocardiográficas mais comuns, consequentes das alterações cardíacas induzidas por esportes de alto desempenho e altos níveis de treinamento, incluem-se a bradicardia sinusal e o bloqueio AV. Habitualmente, não requerem cuidados ou atenção especial desde que sejam assintomáticas ou não produzam pausas superiores a 4 segundos. O bloqueio AV de 1º grau é mais habitual, seguido do bloqueio AV de 2º grau tipo Mobitz I. Já o tipo II e o bloqueio atrioventricular de 3º grau são achados mais incomuns, mesmo em atletas, e devem ser considerados como um sinal de possíveis lesões orgânicas.

A ocorrência de formas ventriculares complexas de arritmia deve sempre levar a exame cardiológico em busca de substrato cardiogênico, especialmente a cardiomiopatia hipertrófica ou dilatada. A presença de arritmias ventriculares sem evidência de doença cardíaca subjacente parece não indicar um risco especial ou aumentado de morte cardíaca súbita. Maior incidência de hipertrofia ventricular direita e/ou esquerda, elevação do segmento ST (repolarização precoce) e alterações lábeis da onda T, ambas reversíveis ao exercício e consideradas fisiológicas no ECG de atletas.

O treinamento físico de *endurance* ou de grande monta expõe o coração a intensas sobrecargas ao longo do tempo. Essas constantes exposições ao treinamento intenso podem gerar alterações no automatismo cardíaco como descrito, além das alterações da condução atrioventricular, despolarização e repolarização ventricular.^1 , 2 , 6^

Adicionalmente, esses ajustes estruturais cardíacos podem ser marcantes e levar a aumentos de até 85% na massa do ventrículo esquerdo. Embora essas alterações funcionais e estruturais sejam documentadas, ainda são desconhecidos os seus limites reais dentro de padrões considerados normais, bem como suas consequências em longo prazo.

Stein et al.,^5^ descreveram ações do treinamento de alto rendimento como corolário de seus efeitos no nó sinusal, onde o aumento do tônus parassimpático, a redução do tônus simpático e os componentes não autonômicos podem contribuir para a bradicardia sinusal e adaptações no sistema especial de condução cardíaca. Tais mecanismos levam a maior prevalência de anormalidades na condução intrínseca atrioventricular observada em atletas.

Em atletas de elite, além da predominância do tônus vagal e, consequentemente, da bradicardia em repouso, que aumentam os valores absolutos da duração do intervalo QT,^7 , 8^ um aumento na massa do ventrículo esquerdo é considerado um fenômeno fisiológico benigno, também conhecido como “coração de atleta”. Observações feitas, como intervalo QT ligeiramente prolongado isolado em atletas, podem refletir a repolarização tardia resultante do aumento da espessura da parede ventricular^8 , 9^ e/ou da bradicardia, tanto como reflexo do treinamento quanto eventualmente como forma de comprometimento no sistema de condução especial do estímulo cardíaco.^10 , 11^ Frequentemente, esses atletas de alta demanda apresentam remodelamento do nó AV, caracterizado por vários graus de bloqueio de condução AV, ritmo atrial ou juncional baixo não-sinusal e, mais raramente, bloqueio AV completo.^1 , 2 , 6 , 9^ Esses distúrbios da condução AV dependem do status do condicionamento físico e estão relacionados não apenas ao aumento da atividade parassimpática sobre o nó AV, mas também ao remodelamento secundário das fibras do nó AV e ao acoplamento célula a célula.^8 , 9^

Assim, a análise das contribuições autonômicas para a dependência da variabilidade da duração dinâmica da repolarização ventricular (VRD) pode ser uma ferramenta valiosa para avaliar a adaptação da VRD à duração do ciclo cardíaco nessa população.^12^

Em estudo prévio, Nazario e Benchimol-Barbosa^13^ descreveram a variabilidade da duração da repolarização ventricular batimento a batimento avaliada por fases de aceleração e desaceleração cardíaca em atletas. A duração da repolarização ventricular dinâmica (DRV) e o acoplamento com intervalo RR estão relacionados ao controle autonômico e à estabilidade elétrica do miocárdio. A retificação de fase da série com intervalo RR separa as fases de aceleração e desaceleração, refletindo influências simpáticas e parassimpáticas na frequência cardíaca, respectivamente, onde observaram que esses apresentam maior duração da repolarização ventricular para todas as durações do intervalo RR.

Nos atletas, a variabilidade da DRV diminui à medida que o intervalo RR aumenta, indicando um efeito benéfico da aptidão física na estabilidade da repolarização e também a avaliação do intervalo RR usando a abordagem de início ou pico de onda como pontos fiduciais proporcionando resultados precisos e adequados para a análise da variação fisiológica do intervalo entre os batimentos.^13 , 14^

Embora os distúrbios da condução AV tenham sido documentados repetidamente em atletas, a adaptação dinâmica da condução AV ao ciclo cardíaco nessa população ainda precisa de esclarecimentos. Na população em geral, a duração AV varia dinamicamente de acordo com a duração do intervalo RR, caracterizando um efeito semelhante a uma sanfona. Entretanto, em atletas, o remodelamento autonômico pode influenciar a condução dinâmica AV na adaptação do intervalo RR, levando a um comportamento distinto da condução AV, em resposta à variação do intervalo RR relacionada ao tempo. O estudo de Benchimol-Barbosa et al.^15^ avaliou as variabilidades do tempo de condução AV (TCAV) batimento por batimento e do intervalo RR em corredores de elite e indivíduos sedentários saudáveis, em repouso, com o objetivo de avaliar o efeito do status da aptidão física na duração do acoplamento espontâneo de TCAV ao intervalo RR. Nesse estudo, os atletas apresentaram valores RR médios e desvios-padrão-RR significativamente maiores que os controles e o *slope* RR-TCAV nos controles e atletas resultou em diferenças significativas entre os grupos, demonstrando que essa inclinação do RR-TCAV diminui à medida que a capacidade metabólica (MET) aumenta.

Acreditamos que algum mecanismo de proteção orgânica intrínseca seja ativado quando o indivíduo sofre pelo exercício crônico e intenso essas adaptações fisiológicas e assim desenvolve, através dessa resposta discrepante observada, uma defesa para manutenção da condução cardíaca especializada.

Ainda é necessário investigar o potencial impacto dos achados atuais em contextos clínicos, como um marcador para taquiarritmias supraventriculares, particularmente a arritmia reentrante do nó AV e fibrilação atrial.

Finalmente, observamos que o achado intrigante do estudo de Benchimol-Barbosa^15^ foi justamente a discordância entre o acoplamento dinâmico do TCAV e denotar as respostas distintas entre atletas e sedentários com relação ao comportamento do intervalo PR e RR. Tais observações merecerão maiores investigações e seguimentos sobre os potenciais efeitos do treinamento físico de alta intensidade e aprimoramentos nas orientações clínicas dessa população.
