# Supplementary material for: Distúrbios do Sistema de Condução Atrioventricular e Potenciais Riscos de Eventos Arrítmicos em Atletas de Alta Resistência
Source: Arq Bras Cardiol. 2020 Jul 28;115(1):78–9. [Article in Portuguese] doi: 10.36660/abc.20200668 (PMC8384336; doi:10.36660/abc.20200668)
Supplement: Translation

# Translation: Atrioventricular Conduction System Disorders and Potential Risks of Arrhythmic Events in Endurance Athletes

**Authors:** Carlos Alberto Cordeiro Hossri

**Affiliations:** 1 Brazil Instituto Dante Pazzanese de Cardiologia, São Paulo, SP – Brazil; 2 Brazil Hospital do Coração, São Paulo, SP – Brazil; 3 Brazil HCor - Associação Beneficiente Síria, São Paulo, SP – Brazil

**Keywords:** Keywords: Athletes, Resistance Training, Physical Fitness, Ventricular Remodeling, Sedentarism, Arrhythmias, Cardiac, Eletrocardiography/methods, Ventricular Function

*Short Editorial related to the article: Assessing Dynamic Atrioventricular Conduction Time to RR-interval Coupling in Athletes and Sedentary Subjects*

Some disorders found on electrocardiograms are commonly seen in endurance or high-performance athletes and often have characteristics that are similar to those observed in elderly individuals or in those with cardiovascular disease.^1 - 4^

High-intensity training done by high-performance athletes can induce intrinsic physiological adaptations to the cardiac stimulus conduction system and, consequently, a higher prevalence of abnormalities in atrioventricular (AV) conduction.^3 , 5^

The physiological or even pathophysiological mechanisms by which athletic training induces such intrinsic changes in the cardiac conduction system still have limited understanding, and are likely to be multifactorial. However, the anatomical changes observed, such as atrial and ventricular dilation, demonstrated the creation of a mechanical-electrical remodeling necessary to cause intrinsic AV electrophysiological adaptations.^4 - 6^

Among the most common electrocardiographic expressions, resulting from cardiac changes induced by high-performance sports and high levels of training, include sinus bradycardia and AV block. They do not usually require special care or attention as long as they are asymptomatic or do not produce pauses longer than 4 seconds. First-degree AV block is more common, followed by 2^nd^degree Mobitz I AV block. Mobitz II and 3^rd^degree atrioventricular blocks are more unusual findings, even in athletes, and should be considered a sign of potential organic injuries.

The occurrence of complex ventricular forms of arrhythmia should always lead to cardiological examination in search of cardiogenic substrate, especially hypertrophic or dilated cardiomyopathy. The presence of ventricular arrhythmias with no evidence of underlying heart disease does not appear to indicate any special or increased risk of sudden cardiac death. Higher incidence of right and/or left ventricular hypertrophy, reversible ST-segment elevation on exercise and reversible abnormalities on exercise on T waves (T negativity, sudden and/or excessive T waves) can be considered physiological abnormalities in the athletes’ ECG scans.

Endurance or major physical training exposes the heart to intense overloads over time. These constant exposures to intense training can generate cardiac automatism disorders as described, in addition to atrioventricular conduction disorders, depolarization and ventricular repolarization.^1 , 2 , 6^

Besides, these cardiac structural adjustments can be remarkable and lead to increases of up to 85% in left ventricular mass. Although these functional and structural abnormalities are documented, their actual limits within standards considered normal, as well as their long-term consequences, are still unknown.

Stein et al.^5^ described high-performance training actions as a corollary of their effects on sinus node, where increased parasympathetic tone, reduced sympathetic tone and non-autonomic components can contribute to sinus bradycardia and adaptations to the special system of cardiac conduction. Such mechanisms lead to a higher prevalence of abnormalities in intrinsic atrioventricular conduction observed in athletes.

In elite athletes, in addition to the predominance of vagal tone and, consequently, bradycardia at rest, which increase absolute QT interval duration,^7 , 8^ an increase in left ventricular mass is considered a benign physiological phenomenon, also known as “athlete’s heart”. Observations made, such as a slightly prolonged isolated QT interval in athletes, may reflect the late repolarization resulting from increased ventricular wall thickness^8 , 9^ and/or bradycardia, both as a reflex of training and ultimately as a form of impairment to the special conduction system of the cardiac stimulus.^10 , 11^ These endurance athletes often present AV node remodeling, characterized by varying degrees of AV conduction block, low non-sinus atrial or junctional rhythm and, more rarely, complete AV block.^1 , 2 , 6 , 9^ These AV conduction disorders depend on the fitness status and are related not only to increased parasympathetic activity on the AV node, but also to the secondary remodeling of the AV node fibers and to cell-to-cell coupling.^8 , 9^

Thus, the analysis of autonomic contributions to the dependence of the variability in the dynamic duration of ventricular repolarization (DVR) can be a valuable tool to assess the adaptation of DVR to the cardiac cycle duration in this population.^12^

In a previous study, Nazario and Benchimol-Barbosa^13^ described the variability in the duration of beat-by-beat ventricular repolarization assessed by phases of cardiac acceleration and deceleration in athletes. The duration of dynamic ventricular repolarization (DVR) and coupling with RR interval are related to autonomic control and electrical myocardial stability. The phase rectification of the series with RR interval separates the acceleration and deceleration phases, reflecting sympathetic and parasympathetic influences on heart rate, respectively, where they observed that these have a longer ventricular repolarization duration for all RR interval durations.

In athletes, DVR variability decreases as the RR interval increases, indicating a beneficial effect of physical fitness on the stability of repolarization and also the evaluation of RR interval using a start or peak wave approach as fiducial points providing proper accurate results for the analysis of physiological variation of the beat-to-beat interval.^13 , 14^

Although AV conduction disorders have been repeatedly documented in athletes, the dynamic adaptation of AV conduction to the cardiac cycle in this population still needs clarification. In the general population, AV duration varies dynamically according to the RR interval duration, characterizing an accordion-like effect. However, in athletes, autonomic remodeling can influence dynamic AV conduction in the adaptation of the RR interval, leading to a different behavior from AV conduction, in response to the variation of the RR interval related to time. The study by Benchimol-Barbosa et al.^15^ evaluated beat-by-beat variability of AV conduction time (AVCT) and RR interval in elite runners and in healthy sedentary individuals, at rest, with the objective of evaluating the effect of physical fitness on the duration of spontaneous coupling of AVCT to the RR interval. In this study, athletes had mean RR values and RR standard deviations significantly higher than controls and the RR-AVCT slope on controls and athletes resulted in significant differences between groups, demonstrating that this RR-AVCT slope decreases as metabolic capacity (MET) increases.

We believe that some mechanism of intrinsic organic protection is activated when the individuals suffer, due to chronic intense exercising, these physiological adaptations and develop, through this discrepant response, a defense to maintain specialized cardiac conduction.

It is still necessary to investigate the potential impact of current findings in clinical settings, such as a marker for supraventricular tachyarrhythmias, particularly AV nodal reentry arrhythmia and atrial fibrillation.

Finally, we observed that the intriguing finding in the Benchimol-Barbosa^15^ study was precisely the disagreement between the dynamic coupling of AVCT and denoting the different responses between athletes and sedentary people regarding the behavior of PR and RR intervals. Such observations deserve further investigation and follow-up on the potential effects of high-intensity training and improvements in the clinical guidelines for this population.
